# Effectiveness and Safety of Pangenotypic Regimens in the Most Difficult to Treat Population of Genotype 3 HCV Infected Cirrhotics

**DOI:** 10.3390/jcm10153280

**Published:** 2021-07-25

**Authors:** Dorota Zarębska-Michaluk, Jerzy Jaroszewicz, Anna Parfieniuk-Kowerda, Ewa Janczewska, Dorota Dybowska, Małgorzata Pawłowska, Waldemar Halota, Włodzimierz Mazur, Beata Lorenc, Justyna Janocha-Litwin, Krzysztof Simon, Anna Piekarska, Hanna Berak, Jakub Klapaczyński, Piotr Stępień, Barbara Sobala-Szczygieł, Jolanta Citko, Łukasz Socha, Magdalena Tudrujek-Zdunek, Krzysztof Tomasiewicz, Marek Sitko, Beata Dobracka, Rafał Krygier, Jolanta Białkowska-Warzecha, Łukasz Laurans, Robert Flisiak

**Affiliations:** 1 Department of Infectious Diseases, Jan Kochanowski University, 25-317 Kielce, Poland; p_stepien@interia.pl; 2 Department of Infectious Diseases and Hepatology, Medical University of Silesia, 40-055 Katowice, Poland; jerzy.jr@gmail.com (J.J.); sobala.szczygiel@op.pl (B.S.-S.); 3 Department of Infectious Diseases and Hepatology, Medical University of Białystok, 15-540 Białystok, Poland; anna.parfieniuk@gmail.com (A.P.-K.); robert.flisiak1@gmail.com (R.F.); 4 Department of Basic Medical Sciences, Faculty of Health Sciences in Bytom, Medical University of Silesia, 41-902 Bytom, Poland; e.janczewska@poczta.fm; 5 Department of Infectious Diseases and Hepatology, Collegium Medicum, Nicolaus Copernicus University, 87-030 Bydgoszcz, Poland; d.dybowska@wsoz.pl (D.D.); mpawlowska@cm.umk.pl (M.P.); w.halota@wsoz.pl (W.H.); 6 Clinical Department of Infectious Diseases, Medical University of Silesia in Katowice, 41-500 Chorzów, Poland; wlodek.maz@gmail.com; 7 Pomeranian Center of Infectious Diseases, Medical University Gdańsk, 80-214 Gdańsk, Poland; lormar@gumed.edu.pl; 8 Department of Infectious Diseases and Hepatology, Medical University Wrocław, 50-367 Wrocław, Poland; justynajanocha@o2.pl (J.J.-L.); krzysimon@gmail.com (K.S.); 9 Department of Infectious Diseases and Hepatology, Medical University of Łódź, 90-419 Łódź, Poland; annapiekar@gmail.com; 10 Hospital for Infectious Diseases in Warszawa, 02-091 Warsaw, Poland; hberak@wp.pl; 11 Department of Internal Medicine and Hepatology, Central Clinical Hospital of the Ministry of Internal Affairs and Administration, 00-241 Warszawa, Poland; klapaj@gmail.com; 12 Medical Practice of Infections, Regional Hospital, 10-561 Olsztyn, Poland; citkoj@wss.olsztyn.pl; 13 Department of Infectious Diseases, Hepatology and Liver Transplantation, Pomeranian Medical University, 71-455 Szczecin, Poland; theville@wp.pl (Ł.S.); asklepiada@wp.pl (Ł.L.); 14 Department of Infectious Diseases, Medical University of Lublin, 20-059 Lublin, Poland; magdalena.tudrujek@gmail.com (M.T.-Z.); tomaskdr@poczta.fm (K.T.); 15 Department of Infectious and Tropical Diseases, Jagiellonian University, 31-088 Kraków, Poland; sitkomar@o2.pl; 16 MED-FIX, 53-522 Wrocław, Poland; dobrackab@gmail.com; 17 Outpatients Hepatology Department, State University of Applied Sciences, 62-510 Konin, Poland; rafalkrygier@gmail.com; 18 Department of Infectious and Liver Diseases, Medical University Łódź, 90-419 Łódź, Poland; jbialkowska@pro.onet.pl; 19 Multidisciplinary Regional Hospital, 66-418 Gorzów Wielkopolski, Poland

**Keywords:** hepatitis C, genotype 3, liver cirrhosis, pangenotypic

## Abstract

There is still limited data available from real-world experience studies on the pangenotypic regimens in patients with genotype (GT) 3 hepatitis C virus (HCV) infection and liver cirrhosis. The current study aimed to evaluate the efficacy and safety of pangenotypic regimens in this difficult-to-treat population. A total of 236 patients with mean age 52.3 ± 11.3 years and male predominance (72%) selected from EpiTer-2 database were included in the analysis; 72% of them were treatment-naïve. The majority of patients (55%) received the combination of sofosbuvir/velpatasvir (SOF/VEL), 71 without and 58 with ribavirin (RBV), whereas the remaining 107 individuals were assigned to glecaprevir/pibrentasvir (GLE/PIB). The effectiveness of the treatment following GLE/PIB and SOF/VEL regimens (96% and 93%) was higher compared to SOF/VEL + RBV option (79%). The univariate analysis demonstrated the significantly lower sustained virologic response in males, in patients with baseline HCV RNA ≥ 1,000,000 IU/mL, and among those who failed previous DAA-based therapy. The multivariate logistic regression analysis recognized only the male gender and presence of ascites at baseline as the independent factors of non-response to treatment. It should be emphasized that despite the availability of pangenotypic, strong therapeutic options, GT3 infected patients with cirrhosis still remain difficult-to-treat, especially those with hepatic impairment and DAA-experienced.

## 1. Introduction

Chronic infection with the hepatitis C virus (HCV) seems to be one of the significant health problems worldwide. Approximately 71 million people are affected globally, of whom 400,000 died annually due to the consequences of the disease [1]. The most severe complications of chronic hepatitis C (CHC) with a risk of death are liver cirrhosis and hepatocellular carcinoma (HCC). The development of liver fibrosis leading to cirrhosis occurs in nearly 20% of patients, and, on average, two decades of HCV infection are needed for this [2]. However, the rate of progression of fibrosis varies between different patients and depends on both viral and host predictors [2]. Male gender, the age of infection over 40 years, coinfection with hepatitis B virus (HBV) and human immunodeficiency virus (HIV), obesity, alcohol abuse are listed among variables related to the infected person, whereas the most important viral predictor for the accelerated fibrosis is genotype (GT) 3 HCV, which is second in frequency worldwide accounting for 25–30% all HCV cases [3,4,5,6]. In the era of interferon (IFN) based therapy, patients with liver cirrhosis had limited access to antiviral treatment due to safety issues and low effectiveness [7]. The implementation of the IFN-free DAA regimens has removed those safety-related limitations, but sofosbuvir (SOF) plus ribavirin (RBV), the only option available initially for GT3 patients, had still relatively low efficacy as compared to the cure rate achieved with DAA therapies in other GTs-infected individuals and treatment with daclatasvir (DCV) plus SOF was not available worldwide [8,9,10]. Therefore, at the beginning of the IFN-free era, cirrhotics infected with GT3 were assumed to be the most difficult-to-treat patients with CHC. The latest development in the antiviral treatment of this subpopulation was the registration of pangenotypic regimens. According to the recent guidelines, two options are recommended in patients with liver cirrhosis in the course of GT3 infection, the combination of protease inhibitor glecaprevir (GLE) with the inhibitor of non-structural protein 5A (NS5A) pibrentasvir (PIB), and SOF, polymerase inhibitor with velpatasvir (VEL), acting by inhibition of NS5A HCV [11,12,13,14]. However, available data in this population are based on limited studies, which usually included a small number of patients. The current study aimed to evaluate the efficacy of pangenotypic regimens in patients with liver cirrhosis infected with GT3 in the real-world experience.

## 2. Materials and Methods

The analyzed population consisted of CHC patients with liver cirrhosis infected with GT3 HCV selected from EpiTer-2 database. This sizeable national project supported by the Polish Association of Epidemiologists and Infectiologists includes 13,554 individuals treated with DAA regimens in 22 Polish hepatology centers between 1 July 2015 and 31 December 2020. Clinical data, including the severity of liver disease, the presence of the extrahepatic manifestations, coexisting medical conditions, concomitant medications, coinfections, the history of previous antiviral treatment and currently used regimen, and laboratory parameters were collected at baseline.

The severity of liver disease was assessed based on the non-invasive fibrosis evaluation either by transient elastography (TE) or shear-wave elastography (SWE), and cirrhosis was diagnosed according to recommendations of the European Association for the Study of the Liver (EASL) if liver stiffness ≥13 kilopascals corresponding to a METAVIR score of F4 [11]. In addition, cirrhotic patients were assessed for the oesophageal varices, past or present hepatic decompensation, history of liver transplantation, and scored in Child-Pugh (CP) scale and Model of End Stage Liver Disease (MELD).

HCV RNA was measured at baseline, at the end of treatment (EOT), and 12 weeks after therapy completion. The efficacy endpoint was sustained virologic response (SVR) defined as undetectable HCV RNA post-treatment week 12. The intent-to-treat (ITT) population included all patients who initiated the treatment, whereas per-protocol (PP) analysis was performed after excluding lost follow-up patients considered to be a non-virologic failure. Safety data in terms of adverse events (AE) and deaths were collected during the treatment course and in the 12-weeks follow-up period. Data were collected retrospectively and submitted by an online questionnaire administered by Tiba sp. z o.o.

### Statistical Analysis

Results were expressed as mean (SD) or number (percentage). A P value less than 0.05 was considered significant. The significance of differences was calculated by the χ2 or Fisher exact tests for nominal variables and by the Mann–Whitney test and the Kruskal-Wallis analysis of variance for continuous variables. Univariable comparisons were calculated using the GraphPad Prism 5.1 software (GraphPad Software, Inc., La Jolla, CA, USA). The general logistic regression model was performed with SVR as the dependent variable. Among independent variables tested for the best model were age, sex, response to previous therapy, anamnesis of hepatic decompensation, baseline ascites, serum bilirubin, albumin, platelets, and HCV RNA. Logistic regression models were calculated by use of Statistica 13.0 (TIBCO Software Inc., Palo Alto, CA, USA).

## 3. Results

A total of 236 patients with liver cirrhosis infected with GT3 with mean age 52.3 ± 11.3 years and male predominance (72%) treated with pangenotypic regimens were included in the analysis. One hundred and seven (45%) were assigned to GLE/PIB, whereas the remaining 129 patients received SOF/VEL including 58 on the regimen with RBV. The choice of the therapeutic option was made by treating physicians in line with guidelines of the Polish Group of Experts for HCV and the recommendations of the National Health Fund, taking into consideration patients’ characteristics and drug labels.

No significant differences in demographic variables, as well as rates of comorbidities and concomitant medications, were observed between patients treated with two pangenotypic regimens (Table 1).

Significantly higher bilirubin concentration, lower albumin level, and platelet count were found among patients treated with SOF/VEL + RBV. In addition, in this subpopulation, a significantly higher percentage of those with past and present hepatic decompensation were observed, and a higher rate of individuals in category B of the Child-Pugh scale (Table 2).

The significantly lower percentage of patients treated with SOF/VEL+RBV were treatment-naïve as compared to SOF/VEL and GLE/PIB regimens, 55.2%, 77.5%, and 77.6%, respectively. The relapse rate was the highest among those assigned to SOF/VEL + RBV option, and SOF + RBV was the most frequently used previous regimen in all subpopulations. A total of 30 patients were nonresponders to previous DAA-containing therapy without IFN, and eight of them were treated in the past with NS5A inhibitors. Six of those who previously failed NS5A-containing regimens were treated with SOF/VEL + RBV; the remaining two patients received GLE/PIB in re-therapy.

The majority of patients on the GLE/PIB option received a 12-weeks regimen (60.7%); more than half (55%) of those assigned to SOF/VEL therapy were treated for 12 weeks without RBV (Table 3).

**Figure 1:**
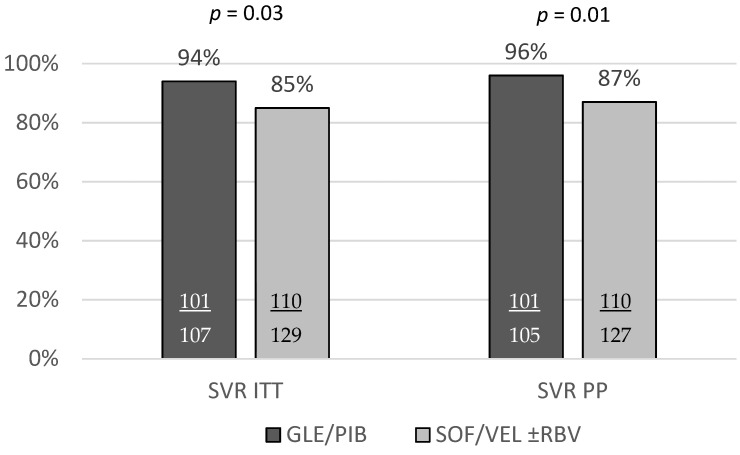
The comparison of the SVR rates between GT3 HCV infected patients with liver cirrhosis treated with GLE/PIB and SOF/VEL ± RBV regimens.

A total of 211 patients achieved an SVR corresponding to 89.4% in the ITT analysis, and after exclusion of four patients lost to follow-up, 91% in the PP analysis. The SVR rate was significantly higher among patients treated with GLE/PIB compared to those receiving SOF/VEL ± RBV both in ITT and PP analyses, 94.4% vs. 85.3% (*p* = 0.03), and 96.2% vs. 86.6%, (*p* = 0.01), respectively (Figure 1).

**Figure 2:**
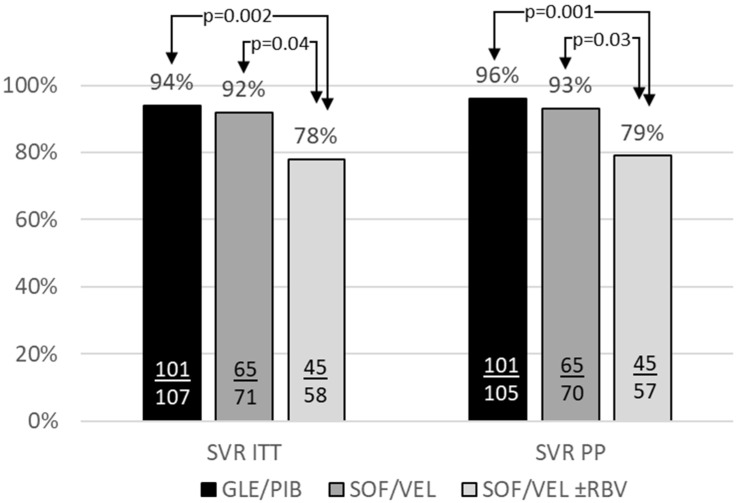
The effectiveness of the GLE/PIB, SOF/VEL, and SOF/VEL + RBV options in GT3 infected patients with liver cirrhosis.

The detailed comparison of an SVR rates revealed no significant difference between GLE/PIB and SOF/VEL regimens, whereas cirrhotics on SOV/VEL + RBV option had significantly lower SVR as compared to both remaining options, 77.6% vs. 91.5% (*p* = 0.04), vs. 94.4% (*p* = 0.002), and 78.9% vs. 92.9% (*p* = 0.003), vs. 96.2% (*p* = 0.001), in ITT and PP analysis, respectively (Figure 2).

A total of twenty-three virologic failures were documented, 6 on GLE/PIB and 17 on SOF/VEL ± RBV regimen (Table 4 and Table 5).

All of them were scored as category A on the CP scale; one experienced RBV dose reduction, and another one discontinued therapy by his own decision. Twenty-one of them were males, and nine were nonresponders to previous DAA-containing therapy, of whom two were treated in the past with pegylated IFN alpha (pegIFNα) + RBV + SOF, 4 received SOF + RBV, two another with GLE/PIB and one patient as a participant of the clinical trial did not respond to uprifosbuvir + grazoprevir + elbasvir/ruzasvir.

A significantly higher rate of males (91.3% vs. 69.4%, *p* = 0.03) was documented in GT3-infected nonresponders to pangenotypic regimens than those who achieved an SVR (Table 6).

The univariate analysis demonstrated the significantly lower SVR in males, in patients with baseline HCV RNA ≥ 1,000,000 IU/mL compared to <1,000,000 IU/mL, and among those who failed previous DAA-based therapy (Table 7).

The multivariate logistic regression analysis recognized the male gender and presence of ascites at baseline as the independent factors of non-response to pangenotypic treatment (Table 8).

The majority of patients completed the treatment course according to schedule, 98.2% in GLE/PIB and 93% in SOF/VEL ± RBV, 6.2% of patients receiving RBV experienced dose modification, three patients discontinued treatment, two due to adverse events (AE), and one by his own decision. A similar proportion of patients in both subpopulations reported at least one AE, with the most common pruritus/skin changes in the course of GLE/PIB treatment and weakness/fatigue during SOF/VEL ± RBV therapy (Table 9).

Three serious AE in patients treated with SOF/VEL + RBV, but not related to this regimen, were documented. In addition, seven AEs of particular interest related to the deterioration of the liver function were reported, including ascites in 4 patients, gastrointestinal bleeding in 2 individuals, and hepatic encephalopathy in one person.

## 4. Discussion

After more than four years elapsed since the registration of the highly potent pangenotypic regimens, the published data from real-world experience (RWE) studies on the use of these medications in GT3 infected patients with liver cirrhosis are still limited, and most of them included a small number of patients.

The single tablet SOF/VEL combination was the first available highly effective option registered for patients with CHC regardless of the HCV genotype, the history of previous therapy, and liver fibrosis. For those with GT3 infection and liver cirrhosis, a 12-week treatment duration was approved based on the results of clinical trials demonstrating cure rates of 91–93%, which is comparable to 93% reported in our analysis [15,16,17]. The better efficacy of 97.5% was achieved in RWE analysis performed by Mangia et al. among 205 Italian GT3 infected patients with liver cirrhosis despite the higher percentage of CP B patients compared to our cohort [18]. However, it should be noted that no DAA-experienced patients were included in the study in contrast to our analysis. The population treated with SOF/VEL in 16 clinical practice cohorts worldwide comprising also DAA-experienced individuals except NS5A-containing regimens achieved an SVR of 93% (332/356) [19]. On the other hand, the cure rate following the SOF/VEL option reported among the RWE cohort of American Veterans, including previously untreated and those who received both IFN- and DAA-based regimens, was 86.5%, lower compared to our result [20].

Even lower efficacy of 79% was achieved in the current analysis in patients treated with SOF/VEL and RBV. It should be noted that the addition of RBV is an option to consider in compensated cirrhotics infected with GT3, whereas it is recommended in the case of decompensated individuals for whom the SOF/VEL is the only registered DAA pangenotypic regimen [21]. The differences in baseline characteristics of patients with a significantly higher number of those with more severe liver disease and the higher rate of treatment-experienced ones among individuals receiving therapy with RBV seem to be the difference of great importance that affects the effectiveness of the treatment with SOF/VEL regimen. Our findings on lower SVR with the SOF/VEL + RBV regimen contradict the results of clinical trials with 96% cure rates, but both studies included only IFN-based treatment-experienced individuals [16,17].

The SVR rate of 95.5% (192/201) was reported for SOF/VEL + RBV option in analysis from multinational RWE presented by Fagiuoli et al., but the range was between 88% and 100% [19]. Mangia et al. documented a 90.5% cure rate with SOF/VEL + RBV regimen in the RWE population, but only ten GT3 infected patients with liver cirrhosis were included [22]. The efficacy of 88% was demonstrated in a real-life population consisted of 34 patients, including 31 treatment-experienced with both IFN- and DAA-based except NS5A-containing regimens [23]. The much more numerous RWE cohort comprising 267 cirrhotic American Veterans treated with SOF/VEL + RBV analyzed by Belperio et al., including NS5A-experienced individuals, responded in 84.5% [20]. Since the failure of prior antiviral therapy, especially DAA containing antiviral therapy, is well recognized as a negative predictor of SVR, the low efficacy documented in our analysis may be influenced by a high percentage of nonresponders in the SOF/VEL + RBV arm, 26/58 (45%), with of whom 21 were treated with DAA [24]. Nine of them received a longer therapy duration 24 weeks, seven responded to treatment, and one was lost to follow-up, giving an 87.5% SVR rate in PP analysis. According to the label, the longer treatment course of SOF/VEL + RBV may be considered in patients who have failed therapy with an NS5A-containing regimen based on analysis from phase 2 and 3 clinical trials. However, there are no clinical data to support this recommendation [21,25]. Therefore further studies are needed to clarify the need for ribavirin in the treatment of decompensated genotype 3 infected cirrhotics who failed previous DAA-based therapy. In the current analysis, six of eight NS5A-experienced patients were treated with SOF/VEL + RBV; three of them failed to achieve an SVR, two with 12-week and another with a 24-week regimen. The remaining two NS5A-experienced patients underwent successful treatment with a 16-week GLE/PIB regimen; however, the numbers are too small to draw conclusions.

The dual therapy of GLE/PIB was approved for GT3 infected patients with compensated liver cirrhosis, and initially, a 12-week option was recommended for treatment-naïve and a 16-week regimen for treatment-experienced individuals based on the results from the clinical trials [26]. With the update of the label made upon the findings from the EXPEDITION-8 trial treatment-naïve, GT3 infected cirrhotics received the possibility to shorten the therapy length to 8 weeks without losing efficacy [27]. In our analysis, the majority of treatment-naïve patients were assigned to a 12-week regimen with an efficacy rate of 97%, while treatment-experienced individuals responded in 95% to 16-week therapy, which is comparable to 98% and 96% SVR rates documented in SURVEYOR-II part 3 study [28]. The data pooled from five phase 2 and 3 clinical trials, including a total of 120 patients with compensated liver cirrhosis, documented a 97% efficacy rate in treatment-naïve following 12-week therapy and 94% as a result of 16-week regimen in treatment-experienced patients [29]. A higher cure rate of 100% was reported in 12 cirrhotic patients from the German Hepatitis-C Registry receiving GLE/PIB, and among Italian cirrhotics treated for 12 or 16 weeks depending on the history of previous treatment, but no precise information on the number of patients, in this case, was added [30,31]. A lower SVR of 83% was demonstrated in 6 treatment-naïve cirrhotic GT3 infected individuals by Toyoda et al. [32]. Very limited RWE data based on small numbers of patients are available for treatment-naïve GT3 infected patients with liver cirrhosis treated with GLE/PIB for 8 weeks. The first published paper from the USA reported a 100% response rate in a group of 4 patients [33]. The same effectiveness was documented by Lampertico et al. following the 8-week GLE/PIB treatment duration in 19 cirrhotic patients with GT3 infection from seven small RWE studies included in the summary analysis [34,35]. A much lower SVR of 72% in PP analysis was demonstrated in nine GT3 infected cirrhotics in our previous study from the EpiTer-2 database, but it was due to a small subset of patients [36]. In the current study, 16 patients treated for 8 weeks achieved SVR, which gives an unsatisfactory rate of 84% in PP analysis, lower than demonstrated for a 12-week regimen with statistical significance for ITT analysis (80% vs. 95.4%, *p* = 0.05), however, it should be noted that a number of patients on 8-week regimen was still low. Further investigations in a large population of GT3 infected cirrhotics are needed to assess the real-world efficacy of an 8-week GLE/PIB regimen. According to label glecaprevir as a protease inhibitor included in the glecaprevir/pibrentasvir regimen is not recommended in moderate hepatic impairment (Child-Pugh B), and is contraindicated in Child-Pugh C patients only. Our study did not include patients with Child-Pugh C and only 4.7% of those treated with glecaprevir/pibrentasvir were classified as Child-Pugh B. The decision to use a protease inhibitor (glecaprevir) in a patient with Child-Pugh B was made by the treating physician.

According to the best of our knowledge, only two available studies made a direct comparison between different pangenotypic regimens in GT3 infected patients, including those with compensated liver cirrhosis. One of them is the analysis performed among 76 Spanish patients with GT3 infection, of whom 12 were diagnosed as cirrhotics, nine were treated with SOF/VEL, including three receiving RBV additionally, and three were assigned to GLE/PIB. The reported efficacy rates were 89% for SOF/VEL ± RBV (8/9) regimen and 67% (2/3) for GLE/PIB option [37]. The second available RWE study comparing SOF/VEL ± RBV, GLE/PIB, and SOF/DCV regimens in GT3 infected patients was made by Soria et al. in a multicentre cohort of Italian patients [38]. Ninety-nine of 2082 individuals included in the study had liver cirrhosis, and despite the difference in SVR rates with 100% in 21 patients treated with GLE/PIB and 93.6% among 78 those receiving SOF/VEL ± RBV regimen, no statistical significance was demonstrated. The comparative analysis concerning demographic, clinical, and laboratory variables between two cirrhotic subpopulations was not provided since the primary comparison was performed among GT3 patients regardless of the liver fibrosis.

No specific safety issues were observed during the treatment course, and we confirmed comparable tolerability across regimens with only a higher rate of RBV-related anemia in SOF/VEL ± RBV. Our findings are in line with the results of clinical trials and RWE studies [15,16,17,18].

The several limitations of the current study related to its real-world nature and retrospective observational design could be identified. Firstly, some clinical data might have been under-reported, including mild adverse events, the prevalence of comorbidities, and concomitant medications usage. No drug monitoring during the therapy hampers the assessment of compliance and its impact on the treatment efficacy. Electronic data capture might result in possible data entry errors. No resistance-associated substitutions (RAS) in previously DAA-nonresponders were tested at baseline. The choice of a therapeutic regimen in all patients was based on the treating physician’s decision regarding recommendations and regulations. However, according to the most recent EASL guidelines, if resistance testing is available and performed, only DAA-experienced patients with the NS5A Y93H RAS at baseline should be treated with SOF/VEL plus RBV, whereas those without should receive SOF/VEL alone, so we assumed that this factor did not affect efficacy reported in our analysis, no NS5A-experienced patient was treated with SOL/VEL [11,39]. Noteworthy, the other regimen prescribed in GT3 infected patients with the presence of Y93H RAS is the combination of SOF/VEL and protease inhibitor voxilaprevir is not recommended in decompensated cirrhotics; moreover, it was not available in Poland within a reimbursed therapeutic program in the analyzed period. Furthermore, finally, since the possibility for a shorter 8-weeks treatment course with GLE/PIB in treatment-naïve GT3 infected patients with liver cirrhosis has emerged very recently, the subset of this population in our analysis is relatively small. However, the study’s major strength is collecting data from the real-world, heterogeneous population representative of routine practice. Moreover, in this study, we included a high number of patients with a low rate of those lost to follow-up (<2%).

## 5. Conclusions

In summary, we confirmed the overall high effectiveness and safety of pangenotypic regimens in the real-world setting of cirrhotics with chronic genotype 3 HCV infection. The highest effectiveness was achieved in those treated with the GLE/PIB regimen, but it was suboptimal if therapy was carried out for 8 weeks. The addition of ribavirin to the SOF/VEL regimen was associated with significantly decreased effectiveness. However, it was related to hepatic decompensation at baseline and failure of previous DAA-based therapy, which are currently indications for ribavirin coadministration. Further studies are needed to clarify the real need for ribavirin in such a difficult-to-treat population of patients treated with SOF/VEL.

## Figures and Tables

**Table 1:** Baseline characteristics of GT3 HCV infected patients with liver cirrhosis treated with pangenotypic regimens.

Parameter	GLE/PIB *n* = 107	SOF/VEL *n* = 71	SOV/VEL + RBV *n* = 58	*p*
Gender, females/males, *n* (%)	30 (28)/77 (72)	23 (32.4)/48 (67.6)	13 (22.4)/45 (77.6)	0.45
Age [years] mean (SD)	51.8 (10.6)	53.2 (12.5)	53.0 (11.3)	0.96
BMI mean (SD)	27.8 (4.7)	27.5 (4.8)	29.0 (5.6)	0.31
Comorbidities, *n* (%)	75 (70.1)	50 (70.4)	40 (69)	0.98
Concomitant medications, *n* (%)	70 (65.4)	47 (66.2)	45 (77.6)	0.24
ALT IU/L, mean (SD)	141 (116)	132 (92)	106 (70)	0.17
Bilirubin mg/dL, mean (SD)	1.0 (0.6)	0.8 (0.4)	1.3 (0.8)	0.003
Albumin g/dL, mean (SD)	3.9 (0.5)	3.9 (0.5)	3.7 (0.5)	0.02
Creatinine mg/dL, mean (SD)	0.9 (0.6)	0.8 (0.2)	0.8 (0.2)	0.74
Hemoglobin g/dL, mean (SD)	14.4 (1.8)	14.5 (1.5)	13.9 (1.7)	0.27
Platelets, ×1000/µL, mean (SD)	139 (82)	128 (54)	95 (53)	<0.001
HCV RNA × 10^6^ IU/mL, mean (SD)	2.17 (4.31)	1.45 (1.79)	1.49 (2.29)	0.62

HCV, hepatitis C virus; GLE, glecaprevir; PIB, pibrentasvir; SOF, sofosbuvir; VEL, velpatasvir; RBV, ribavirin; SD, standard deviation; BMI, body mass index; ALT, alanine transaminase; HCV RNA, ribonucleic acid of hepatitis C virus.

**Table 2:** Characteristics of the liver disease in GT3 HCV infected patients with liver cirrhosis treated with pangenotypic regimens.

Parameter	GLE/PIB *n* = 107	SOF/VEL *n* = 71	SOF/VEL + RBV *n* = 58	*p*
History of hepatic decompensation, *n* (%)				
Number of patients	2 (1.8)	3 (4.2)	9 (15.5)	0.001
Ascites	1 (0.9)	3 (4.2)	9 (15.5)	<0.001
Encephalopathy	1 (0.9)	1 (1.4)	1 (1.7)	0.91
Documented esophageal varices, *n* (%)	22 (20.6)	11 (15.5)	12 (20.7)	0.66
Hepatic decompensation at baseline, *n* (%)				
Moderate ascites—responded to diuretics	0	1 (1.4)	6 (10.3)	<0.001
Tense ascites—not responded to diuretics	0	0	0	na
Encephalopathy	0	0	0	na
HCC history, *n* (%)	4 (3.7)	2 (2.8)	1 (1.7)	0.76
OLTx history, *n* (%)	0	0	0	na
Child-Pugh, *n* (%)				
A	102 (95.3)	70 (98.6)	53 (91.4)	0.15
B	5 (4.7)	1 (1.4)	5 (8.6)	0.15
C	0	0	0	na
MELD, *n* (%)				
<15	100 (93.6)	67 (94.4)	58 (100)	0.15
15–18	3 (2.8)	1 (1.4)	0	na
19–20	2 (1.8)	1 (1.4)	0	na
>20	1 (0.9)	0	0	na
No data	1 (0.9)	2 (2.8)	0	na
HBV coinfection (HBsAg+), *n* (%)	2 (1.8)	3 (4.2)	0	0.24
HIV coinfection, *n* (%)	7 (6.5)	9 12.7)	3 (5.1)	0.22

HCV, hepatitis C virus; GLE, glecaprevir; PIB, pibrentasvir; SOF, sofosbuvir; VEL, velpatasvir; RBV, ribavirin; hepatocellular carcinoma; OLTx, orthotopic liver transplantation; MELD, Model End-Stage Liver Disease; HBV, hepatitis B virus; HBsAg+, hepatitis B surface antigen; HIV, human immunodeficiency virus.

**Table 3:** Previous and current treatment characteristics of GT3 HCV infected patients with liver cirrhosis treated with pangenotypic regimens.

Parameter	GLE/PIB *n* = 107	SOF/VEL *n* = 71	SOF/VEL + RBV *n* = 58	*p*
History of previous therapy, *n* (%)				
Treatment-naïve	83 (77.6)	55 (77.5)	32 (55.2)	0.004
Nonresponder	3 (2.8)	3 (4.2)	4 (6.9)	0.46
Relapser	16 (14.9)	12 (16.9)	20 (34.5)	0.008
Discontinuation due to safety reasons	0	0	1 (1.7)	na
Unknown type of response	5 (4.7)	1 (1.4)	1 (1.7)	0.37
Previous regimen in patients with treatment failure, *n* (%)	*n* = 24	*n* = 16	*n* = 26	
PegIFNα + RBV	5 (20.8)	6 (37.5)	4 (15.4)	0.24
SOF + PegIFNα + RBV	4 (16.7)	3 (19)	4 (15.4)	0.96
SOF + RBV	12 (50)	7 (43.8)	11 (42.3)	0.85
SOF/VEL ± RBV	2 (8.3)	0	0	na
SOF/LDV	0	0	1 (3.8)	na
GLE/PIB	0	0	4 (15.4)	na
Other	0	0	2 (7.7) *	na
No data	1 (4.2)	0	0	na
Current treatment regimens, *n* (%)				
GLE/PIB, 8 weeks	20 (18.7)	na	na	
GLE/PIB, 12 weeks	65 (60.7)	na	na	
GLE/PIB, 16 weeks	22 (20.6)	na	na	
SOF/VEL, 12 weeks	na	71 (100)	na	
SOF/VEL + RBV, 12 weeks	na	na	48 (82.7)	na
SOF/VEL + RBV, 24 weeks	na	na	10 (16.3)	

HCV, hepatitis C virus; GLE, glecaprevir; PIB, pibrentasvir; SOF, sofosbuvir; VEL, velpatasvir; RBV, ribavirin; PegIFNα, pegylated interferon alpha; LDV, ledipasvir. * IFNα + RBV, Uprifosbuvir + Grazoprevir + Elbasvir/Ruzasvir.

**Table 4:** Characteristics of 6 virologic failures to GLE/PIB regimen.

Patient	Age	CP	Regimen	History of Previous Therapy	Baseline HCV RNA IU/mL	Treatment Course	EOT	Comment (Possible Reason for Non-Response)
Female 1	56	A	GLE/PIB 12	treatment-naive	2,518,022	according to plan	TD	
Male 1	48	A	GLE/PIB 8	treatment-naive	942,000	according to plan	TND	
Male 2	51	A	GLE/PIB 8	treatment-naive	1,621,033	according to plan	TD	
Male 3	52	A	GLE/PIB 8	treatment-naive	1,483,266	according to plan	TND	
Male 4	30	A	GLE/PIB 12	treatment-naive	1,580,000	according to plan	TND	
Male 5	54	A	GLE/PIB 16	relapse (SOF + RBV)	4,030,000	according to plan	TND	DAA failure

GLE, glecaprevir; PIB, pibrentasvir; CP, Child-Pugh scale; HCV RNA, ribonucleic acid of hepatitis C virus; EOT, end of treatment; TD, target detected; TND, target not detected; SOF, sofosbuvir; RBV, ribavirin; DAA, direct-acting antivirals.

**Table 5:** Characteristics of 17 virologic failures to SOF/VEL ± RBV regimen.

Patient	Age	CP	Regimen	History of Previous Therapy	Baseline HCV RNA IU/mL	Treatment Course	EOT	Comment (Possible Reason for Non-Response)
Female 1	44	A	SOF/VEL + RBV 12	treatment-naive	3,560,000	RBV dose reduction	TD	
Male 1	50	A	SOF/VEL 12	relapse (SOF + RBV)	2,190,000	according to plan	TND	DAA failure
Male 2	54	A	SOF/VEL 12	relapse (SOF + RBV)	5279	according to plan	TND	DAA failure
Male 3	49	A	SOF/VEL 12	treatment-naive	1,014,206	according to plan	TD	
Male 4	38	A	SOF/VEL 12	treatment-naive	4,910,000	according to plan	TD	
Male 5	50	A	SOF/VEL 12	treatment-naive	70,000	according to plan	TD	
Male 6	58	A	SOF/VEL + RBV 12	treatment-naive	1,620,000	according to plan	TND	
Male 7	54	A	SOF/VEL + RBV 12	relapse (SOF + RBV)	667,000	according to plan	TND	DAA failure
Male 8	53	A	SOF/VEL + RBV 12	relapse (PR + SOF)	261,902	according to plan	TND	DAA failure
Male 9	29	A	SOF/VEL + RBV 12	relapse (PR + SOF)	534,255	according to plan	TND	DAA failure
Male 10	50	A	SOF/VEL + RBV 12	relapse (Uprifosbuvir + Grazoprevir + Elbasvir or Ruzasvir)	2,230,000	according to plan	TND	DAA failure
Male 11	58	A	SOF/VEL + RBV 12	relapse (GLE/PIB)	1,270,000	according to plan	TND	DAA failure
Male 12	51	A	SOF/VEL + RBV 12	treatment-naive	1,790,000	according to plan	TND	
Male 13	70	A	SOF/VEL + RBV 12	treatment-naive	2,420,000	according to plan	TND	
Male 14	52	A	SOF/VEL + RBV 12	treatment-naive	1,220,000	according to plan	TD	
Male 15	73	A	SOF/VEL + RBV 12	treatment-naive	4,270,000	discontinued	TD	Treatment discontinuation
Male 16	56	A	SOF/VEL + RBV 24	relapse (GLE/PIB)	1,080,000	according to plan	TND	DAA failure

SOF, sofosbuvir; VEL, velpatasvir; RBV, ribavirin; CP, Child-Pugh scale; HCV RNA, ribonucleic acid of hepatitis C virus; EOT, end of treatment; TD, target detected; TND, target not detected; DAA, direct-acting antivirals; PR, PegIFNα + RBV; GLE, glecaprevir; PIB, pibrentasvir.

**Table 6:** Virologic nonresponders vs. responders to pangenotypic regimens.

Parameter	Virologic Nonresponders *n* = 23	Responders *n* = 209	*p*
Gender, females/males, *n* (%)	2 (8.7)/21 (91.3)	64 (30.6)/145 (69.4)	0.03
Age [years] mean (SD)	51.3 (10)	52.8 (11.5)	0.67
BMI mean (SD)	28.8 (4.6)	28.0 (5.1)	0.44
Any comorbidity, *n* (%)	16 (69.6)	147 (70.3)	1.00
Concomitant medications, *n* (%)	18 (78.3)	143 (68.4)	0.47
HBV coinfection (HBsAg+), *n* (%)	0	5 (2.4)	1.00
HIV coinfection, *n*(%)	2 (8.7)	16 (7.7)	0.69
Liver stiffness kPa, mean (SD)	28 (13.3)	28.8 (17.5)	0.71
History of hepatic decompensation, *n* (%)	3 (13)	11 (5.3)	0.15
HCC history, *n* (%)	1 (4.3)	6 (2.9)	0.52
Hepatic decompensation at baseline, *n* (%)	2 (8.7)	5 (2.4)	0.14
Child-Pugh B, *n* (%)	0	10 (4.8)	0.60
Treatment-experienced, *n* (%)	9 (39.1)	54 (25.8)	0.22
IFN-free DAA-experienced, *n* (%)	7 (30.4)	29 (13.9)	0.06
ALT IU/L, mean (SD)	143 (85)	129 (102)	0.24
Bilirubin mg/dL, mean (SD)	1.15 (0.38)	1.0 (0.64)	0.01
Albumin g/dL, mean (SD)	3.87 (0.55)	3.86 (0.49)	0.99
Creatinine mg/dL, mean (SD)	0.85 (0.14)	0.85 (0.45)	0.14
Hemoglobin g/dL, mean (SD)	14 (1.9)	14.3 (1.7)	0.51
Platelets, ×1000/µL, mean (SD)	100 (54)	128 (71)	0.04
HCV RNA ×10^6^ IU/mL, mean (SD)	1.79 (1.33)	1.8 (3.45)	0.03

BMI, body mass index; SD, standard deviation; HBV, hepatitis B virus; HBsAg+, hepatitis B surface antigen; HIV, human immunodeficiency virus; HCC, hepatocellular carcinoma; IFN, interferon; DAA, direct-acting antivirals; ALT, alanine transaminase; HCV RNA, ribonucleic acid of hepatitis C virus.

**Table 7:** Treatment effectiveness in subpopulations.

	**Females, *n* = 66**	**Males, *n* = 170**	***p***
SVR ITT	97% (64/66)	85.3% (145/170)	0.01
SVR PP	97% (64/66)	87.3% (145/166)	0.03
	**HCV RNA < 1,000,000, *n* = 131**	**HCV RNA ≥ 1,000,000, *n* = 105**	
SVR ITT	93.1% (122/131)	82.9% (87/105)	0.02
SVR PP	95.3% (122/128)	83.7% (87/104)	0.004
	**Treatment-experienced, *n* = 66**	**Treatment-naive, *n* = 170**	
SVR ITT	81.8% (54/66)	91.2% (155/170)	0.07
SVR PP	85.7% (54/63)	91.7% (155/169)	0.22
	**DAA-experienced, *n* = 49**	**Treatment-naive, *n* = 170**	
SVR ITT	77.5% (38/49)	91.2% (155/170)	0.02
SVR PP	80.9% (38/47)	91.7% (155/169)	0.06
	**BMI < 30, *n* = 161**	**BMI ≥ 30, *n* = 64**	
SVR ITT	88.2% (142/161)	92.2% (59/64)	0.48
SVR PP	89.9% (142/158)	92.2% (59/64)	0.80

SVR, sustained virologic response; ITT, intent to treat; PP, per protocol; HCV RNA, ribonucleic acid of hepatitis C virus; IFN, interferon; DAA, direct-acting antivirals; SOF, sofosbuvir; BMI, body mass index; The bold represent the same level as gender.

**Table 8:** Baseline factors associated with SVR based on the logistic regression model.

	Estimate of β	SE	t-Stat	*p* Value
(Intercept)			550.76	<0.001
Gender (male)	−0.16	0.07	−2.47	0.01
Baseline ascites (no)	0.17	0.07	2.43	0.03
Previous decompensation (no)	0.04	0.07	0.59	0.55
Response to previous therapy (non-response)	0.04	0.09	0.51	0.61
Response to previous therapy (naive)	0.11	0.09	1.22	0.22
Bilirubin	0.03	0.07	0.34	0.73
Platelets	0.05	0.07	0.71	0.48
HCV RNA	0.02	0.06	0.34	0.73

HCV RNA, ribonucleic acid of hepatitis C virus.

**Table 9:** Safety of GLE/PIB and SOF/VEL ± RBV in GT3 infected patients with liver cirrhosis.

Parameter	GLE/PIB *n* = 107	SOF/VEL ± RBV *n* = 129	*p*
Treatment course, *n* (%)			
according to schedule	105 (98.2)	120 (93)	0.12
modified RBV dosage	Na	8 (6.2)	Na
therapy discontinuation	2 (1.8)	1 (0.8) *	0.59
Patients with at least one AE	24 (22.4)	28 (21.7)	1.00
Most common AEs			
weakness/fatigue	7 (6.5)	12 (9.3)	0.48
gastrointestinal symptoms	4 (3.7)	7 (5.4)	0.76
pruritus/skin changes	8 (7.5)	2 (1.6)	0.05
anemia	0	9 (7)	0.004
Death	0	0	na
Other serious adverse events	0	3 (2.3) **	0.25
AEs leading to treatment discontinuation	2 (1.8) ***	0	0.20
AEs of particular interest			
ascites	2 (1.8)	2 (1.6)	1.00
hepatic encephalopathy	0	1 (0.8)	1.00
gastrointestinal bleeding	0	2 (1.6)	0.50

* patient’s decision; ** hepatic decompensation, HCC, pneumonia; *** worsening of depression, exacerbation of heart failure; GLE, glecaprevir; PIB, pibrentasvir; SOF, sofosbuvir; VEL, velpatasvir; RBV, ribavirin; AE, adverse event.

## Data Availability

Data supporting reported results can be provided upon request from the corresponding author.
